# Acute Superior Mesenteric Thrombosis in a Young Adult With No Traditional Risk Factors: A Case Study

**DOI:** 10.7759/cureus.69364

**Published:** 2024-09-13

**Authors:** Maryam K Abulfateh, Salah Alghanem

**Affiliations:** 1 Department of Emergency Medicine, Bahrain Defence Force, Royal Medical Services, Military Hospital, Riffa, BHR

**Keywords:** computed tomography angiography (cta), intestinal ischemia, psychiatric illnesses, superior mesenteric vein, superior mesenteric vein thrombosis

## Abstract

This is a case report of acute superior mesenteric vein (SMV) thrombosis in a 24-year-old male with history of underlying psychiatric symptoms who had no traditional risk factors. The patient presented with abdominal pain, fever, and constipation. The patient’s worsening symptoms led to an eventual diagnosis via computed tomography (CT) imaging. Management included anticoagulation therapy and multidisciplinary care. This case highlights the importance of considering SMV thrombosis in young adults and suggests a potential link between psychiatric conditions and thrombotic events, as evidenced by this patient’s psychiatric history.

## Introduction

Superior mesenteric vein (SMV) thrombosis, the formation of blood clots in the major vein draining the small intestine, is rare in young adults but more common in older individuals [1]. A systematic review from 1966 to 2002, which included 3,692 patients, found that mesenteric venous thrombosis accounted for 3% of acute mesenteric thrombosis cases [1]. Other reviews suggest that acute mesenteric venous thrombosis accounts for between 2% and 10% of cases [2-4].

Mesenteric venous thrombosis is a multifactorial disorder influenced by various risk factors, including both local intra-abdominal inflammatory processes and a range of heritable and acquired thrombophilias. Common predisposing factors include abdominal masses, such as tumors or pseudocysts that lead to venous compression, and inflammatory processes like acute pancreatitis and diverticulitis. Myeloproliferative disorders, particularly those associated with the JAK-2 V617F mutation, are also significant contributors. Furthermore, conditions such as portal hypertension, cirrhosis, and a personal or family history of venous thromboembolism raise the risk. Some of the most important risk factors are acquired thrombophilias, such as cancer and oral contraceptives, inflammatory bowel disease, mesenteric adenopathy, and viral infections like the flu. Inherited thrombophilias, such as factor V Leiden mutation, prothrombin G20210A mutation, and deficiencies in proteins S and C or antithrombin III, along with activated protein C resistance and antiphospholipid syndrome, further elevate the risk. Endoscopic sclerotherapy, obesity surgery, and the hypercoagulable state associated with severe acute respiratory syndrome coronavirus 2 (SARS-CoV-2) infection, also known as coronavirus disease 2019 (COVID-19), has been identified as contributing factors to the development of mesenteric venous thrombosis [2-8]. SARS-CoV-2 infection (i.e., COVID-19) is associated with hypercoagulability [9-12]. However, the exact cause of SMV thrombosis in young adults often remains unclear, highlighting the need for further research to identify specific risk factors in this population.

Understanding SMV thrombosis in young adults is critical for a variety of reasons. First, the condition presents diagnostic challenges, as its symptoms can mimic other digestive disorders, leading to potential misdiagnosis. Given the diagnostic challenges posed by the symptom similarity between SMV thrombosis and other gastrointestinal disorders, further research is essential. Studies focusing on young adults could lead to the development of improved diagnostic criteria and early detection methods, thereby enhancing timely identification and management of SMV thrombosis. Second, untreated SMV thrombosis can result in severe complications, including intestinal infarction and bowel ischemia, making early diagnosis and treatment essential to prevent serious harm. Third, identifying risk factors specific to young adults is considered important for developing preventive strategies and targeted interventions. This emphasis reflects general clinical practice and the need for age-specific considerations in preventive health. Lastly, research focused on this age group could offer valuable insights into optimal treatment approaches, such as anticoagulation therapy, interventional radiology, and surgical options.

## Case presentation

A 24-year-old male presented to the emergency room with a three-day history of right flank pain and right lower quadrant abdominal pain, accompanied by fever, one episode of vomiting, and four days of constipation. He denied any urinary symptoms, trauma, blood in stool, or testicular pain. The patient had no known medical or surgical conditions, did not smoke or consume alcohol, and did not have a personal or family history of coagulopathy. Two days prior, he had visited another hospital with the same complaint and received symptomatic treatment for acute gastritis, but his symptoms worsened.

On presentation, his vital signs were: temperature 36.9°C, blood pressure 140/94 mmHg, heart rate 75 bpm, respiratory rate 17 breaths/min, oxygen saturation (SpO2) 99% on room air, and random blood sugar 5.3 mmol/L. A physical examination revealed a sick-looking, dehydrated patient with periumbilical and right lower quadrant tenderness; the remainder of the examination was unremarkable.

Laboratory studies included a complete blood count, urea and electrolytes, renal function tests, liver function tests, amylase, lipase, and urinalysis. Urine dipstick showed protein 1+. The patient suffered an acute kidney injury (creatinine 140 umol/L, estimated glomerular filtration rate (eGFR) 54 ml/min/1.73 m2). Other tests were normal. Serum lactate, drawn after starting Ringer lactate infusion, was within normal limits (0.76 mmol/L). No prior laboratory studies were available for comparison.

**Figure 1:**
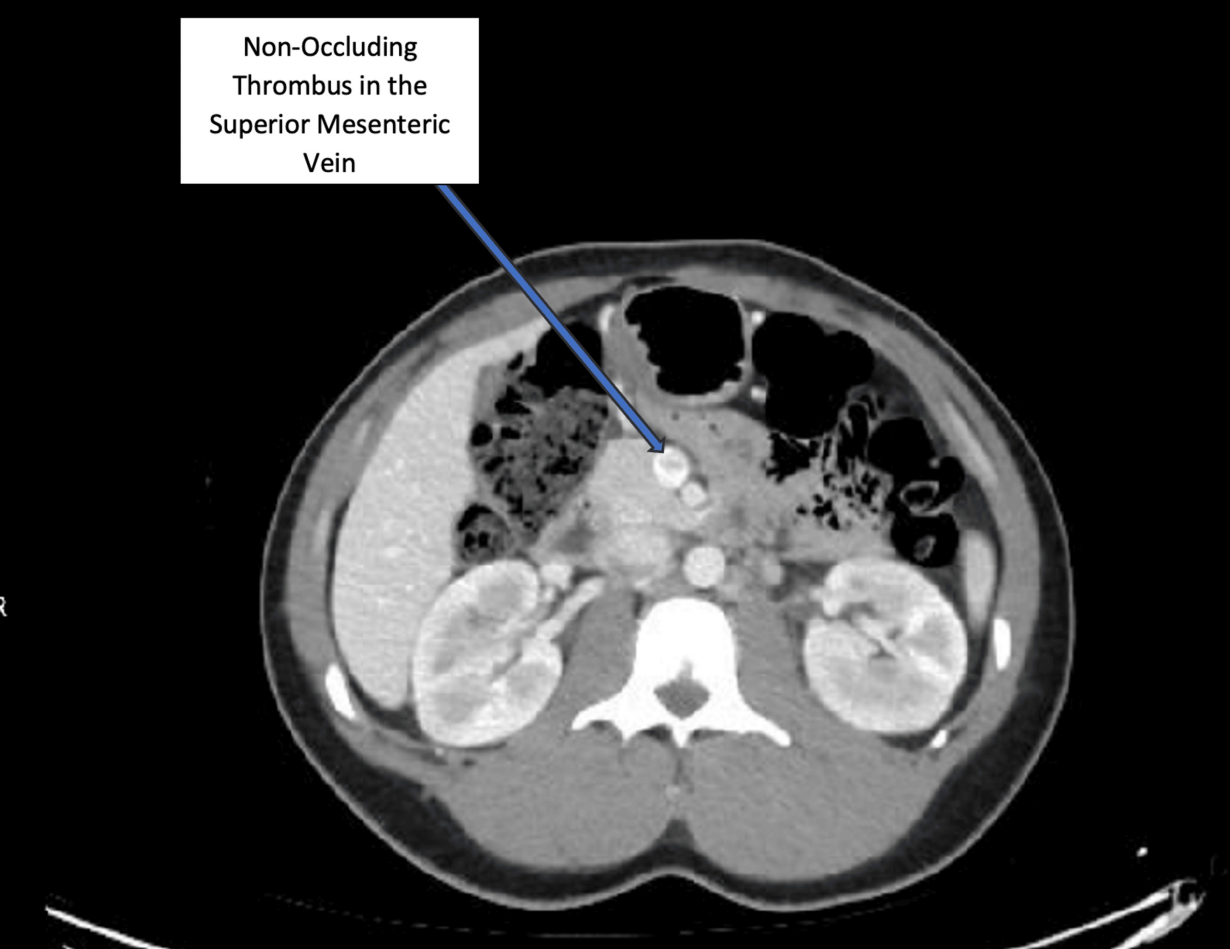
Computed Tomography of the abdomen with intravenous and oral contrast showing non-occluding thrombus in the superior mesenteric vein.

An abdominal radiograph revealed extensive fecal matter accumulation in the colon. Abdominal ultrasound was unremarkable. Due to the severe, disproportionate pain, a computed tomography of the abdomen and pelvis with intravenous (IV) and oral contrast was performed after obtaining high-risk consent due to the patient's acute kidney injury. The scan revealed a non-occluding thrombus in the SMV and jejunal and ileal branches (Figure 1), with normal enhancement in the visualized major abdominal vessels. There was no evidence of infarction, superior mesenteric artery (SMA) thrombus, acute appendicitis, or free fluid.

The patient was admitted under internal medicine care and initially evaluated by gastroenterology and hematology. Due to a complex medical presentation and safety concerns raised by his family, stemming from his history of suicidal ideation and visual hallucinations starting at age 10, psychiatric and neurological consultations were deemed essential. These consultations aimed to address potential underlying psychiatric issues and to rule out neurological conditions. The patient received intravenous fluids, analgesia, lactulose, and initial anticoagulation with enoxaparin 90 mg once daily for four days. Following hematology's recommendation, his treatment was adjusted to apixaban 5 mg twice daily for six months. However, after five days on apixaban, liver function test abnormalities developed, leading to a switch back to enoxaparin and warfarin.

Additional laboratory tests conducted during admission revealed a vitamin D (25-OH) level of 16.3 nmol/L. An iron study was also performed, which revealed normal values. The thrombophilia and antiphospholipid screens both returned negative results, as did the anti-nuclear antibody (ANA) profile. Testing for factor V Leiden and prothrombin gene mutations showed normal results, while anti-beta-2 glycoprotein-1 antibodies, immunoglobulin G and M (IgG, IgM), janus kinase 2 gene (JAK2) mutation, double-stranded DNA (dsDNA) immunoglobulin G antibodies (dsDNS IgG), and anti-cardiolipin antibodies were all negative (Table 1). Stool testing for fecal calprotectin was done to rule out abdominal pathology. Hemoglobin electrophoresis and homocysteine levels were scheduled to be assessed from the next sample.

**Table 1:** Laboratory Results HBsAg: hepatitis B surface antigen; HCV: hepatitis C virus; TP: Treponema pallidum; ANA: anti-nuclear antibody; CTD: connective tissue disease; Ig: immunoglobulin, dsDNS IgG: double-stranded DNA immunoglobulin G; IU/ml: international unit per milliliter; MPL: IgM phospholipid; GPL: IgG phospholipid

Test Name	Titer	Status	Biological Ref. Range	Analytical Method
HIV AB/AG COMBO	0.06	NEGATIVE	1 - 1	Chemiluminescent microparticle immunoassay
HBsAg	0.25	NEGATIVE	1 - 1	Chemiluminescent microparticle immunoassay
Anti-HCV	0.11	NEGATIVE	1 - 1	Chemiluminescent microparticle immunoassay
TP-Antibody (Syphilis)	0.09	NEGATIVE	1 - 1	Chemiluminescent microparticle immunoassay
ANA Screen (CTD)	0.2	NEGATIVE	.7 - 1 Ratio	Fluoroenzyme Immunoassay
dsDNA IgG	1.4	NEGATIVE	10 - 15 IU/ml	Fluoroenzyme Immunoassay
Anti-Cardiolipin IgG	1.5	NEGATIVE	10 - 40 GPL	Fluoroenzyme Immunoassay
Anti-Cardiolipin IgM	1.5	NEGATIVE	10 - 40 MPL	Fluoroenzyme Immunoassay

Magnetic resonance imaging (MRI) and magnetic resonance venography (MRV) brain scans were arranged to exclude organic causes. The brain MRI was unremarkable. There was no evidence of venous sinus thrombosis in the MVR brain.

After five days of intravenous hydration, the patient’s renal functions normalized. He was discharged after 12 days in stable condition on enoxaparin and warfarin, with follow-up appointments with psychiatry and hematology. Enoxaparin was stopped after one month, and warfarin continued for six months with regular lab checks. The patient was considered treated and discharged from the hematology clinic. He continued psychiatric follow-up and was started on aripiprazole (Abilify).

## Discussion

This case highlights a rare occurrence of acute SMV thrombosis in a young adult male with no identifiable traditional risk factors. This case highlights the critical role of imaging in diagnosing SMV thrombosis, especially when symptoms worsen despite initial treatment. It underscores the importance of timely imaging in young patients with unexplained abdominal pain, even when traditional risk factors are not present. This case emphasizes the importance of maintaining a high index of suspicion for SMV thrombosis in young patients with unexplained abdominal pain, even in the absence of traditional risk factors [4]. 

Traditionally, risk factors for SMV thrombosis include conditions like pancreatitis, intra-abdominal malignancies, and thrombophilia, none of which were identified in this patient. Several laboratory tests were conducted, including ones for factor V Leiden and antiphospholipid antibodies, which were negative for both inherited and acquired thrombophilias. Such findings point to the multifactorial nature of SMV thrombosis and highlight the importance of considering non-traditional factors in young, otherwise healthy individuals.

The atypical nature of SMV thrombosis in this demographic challenges conventional understanding of predisposing factors [13]. In a retrospective study at The First Hospital of Hebei Medical University, researchers analyzed data from inpatients with mental illnesses and confirmed venous thromboembolism (VTE) between August 2018 and July 2022. They found that among 12,939 patients with mental illnesses, 1.21% had VTE. The incidence of VTE was highest in patients with organic mental disorders (5.20%) and varied significantly among different mental illnesses. Most VTE cases involved distal deep venous thromboses (79.17%). Higher Hamilton Depression Scale scores were linked to an increased risk of proximal DVT, while higher Hamilton Anxiety Scale scores were protective against DVT progression [14]. Psychiatric illnesses can indeed lead to a prothrombotic state by influencing various physiological mechanisms, including hormonal imbalances caused by chronic stress and unhealthy lifestyle choices such as poor diet and lack of exercise. This understanding aligns with broader research on how stress and mental health impact vascular health and thrombotic risk [15]. 

There is growing evidence that psychiatric disorders, especially those associated with chronic stress, anxiety, and depression, may contribute to a hypercoagulable state. Recent research indicates a significant association between psychiatric disorders and an increased risk of VTE. Patients with remitted major depressive disorder exhibit an enhanced procoagulant state compared to healthy controls [16]. A meta-analysis revealed that psychotic and bipolar disorders are significantly associated with VTE risk, while depression and anxiety disorders show this association in adjusted analyses [17]. A population-based cohort study found that patients with concurrent depressive, bipolar, and schizophrenic disorders have a higher risk of developing deep vein thrombosis and pulmonary embolism compared to the general population [18].

In this patient, it is plausible that the combination of his psychiatric condition and possibly unrecognized subtle prothrombotic factors could have contributed to the development of SMV thrombosis. This underscores the need for further research into the connection between mental health and thrombosis risk, particularly in young adults.

Understanding the interplay between mental health and vascular health is crucial for identifying potential triggers or exacerbating factors for thrombosis in patients with psychiatric illnesses. Considering the lack of traditional risk factors, this case prompts investigation into less common etiologies, such as genetic predispositions or hypercoagulable states associated with psychiatric conditions and thrombotic events. 

Addressing the psychosocial aspects of the patient's care is imperative. Collaborative efforts between hematology, gastroenterology, and psychiatric teams were essential for comprehensive management. The treatment plan should not only focus on anticoagulation and resolving the acute thrombotic episode but also on addressing the psychiatric components, ensuring long-term mental well-being, and reducing the risk of recurrence.

## Conclusions

In conclusion, this case study underscores the importance of a multidisciplinary approach in understanding and managing SMV thrombosis in young patients with psychiatric illnesses and no apparent risk factors. It invites further research into the intricate relationship between mental health and thrombotic events, urging clinicians to broaden their perspectives when encountering such complex clinical scenarios.
